# The relationship between iron deficiency anemia and sexual function and satisfaction among reproductive-aged Iranian women

**DOI:** 10.1371/journal.pone.0208485

**Published:** 2018-12-06

**Authors:** Zahra Nikzad, Mina Iravani, Parvin Abedi, Nahid Shahbazian, Amal Saki

**Affiliations:** 1 Midwifery Department, Reproductive Health Promotion Research Center, Ahvaz Jundishapur University of Medical Sciences, Ahvaz, Iran; 2 Midwifery Department, Menopause Andropause Research Center, Ahvaz Jundishapur University of Medical Sciences, Ahvaz, Iran; 3 Midwifery Department, Menopause Andropause Research Center, Ahvaz Jundishapur University of Medical Sciences, Ahvaz, Iran; 4 Department of Obstetrics and Gynecology, Fertility, Infertility, and Perinatology Research Center, Ahvaz Jundishapour University of Medical Sciences, Ahvaz, Iran; 5 Department of Biostatistics and Epidemiology, School of Public Health, Ahvaz Jundishapur University of Medical Sciences, Ahvaz, Iran; Englewood Hospital and Medical Center, UNITED STATES

## Abstract

Iron deficiency anemia (IDA) is a common micronutrient deficiency worldwide, and an important health problem especially in women of reproductive age. This study aimed to determine the relationship between IDA and sexual satisfaction and function among reproductive-aged Iranian women. In this study, 129 women (52 with IDA and 77 non-IDA) with age 18–45 in Mahshahr, Iran were recruited. Data was gathered by a demographic questionnaire, Female Sexual Function Index (FSFI) and Larson Sexual Satisfaction Questionnaire. Data were analyzed using an independent t-test, Mann-Whitney test, Chi-square, and correlation coefficient test. The results of this study showed that the means of hemoglobin (Hb), hematocrit (HCT), serum iron and ferritin were significantly lower in the IDA group than those in the non-IDA group (p<0.01). All dimensions of sexual function and satisfaction were significantly lower in women with IDA compared to the healthy women (p<0.001). Also, all blood indices for IDA had a significant relationship with all sexual function components and sexual satisfaction (p = 0.01) except for pain with Hb and ferritin. Health care providers should provide screening, education, and counseling about anemia and sexual function in reproductive age women.

## Introduction

Sexual dysfunction is defined as a disorder in the sexual response cycle or pain during sexual intercourse [1]. Almost 40–45% of women suffer from sexual dysfunction [2]. The prevalence of sexual dysfunction reported to be 43% and 31% among women and men in the United States respectively [3]. According to a national survey conducted in Iran, 31.5% of women had sexual dysfunction [4].

Female sexual dysfunction can affect the general health and quality of life [5]. Several factors can cause sexual dysfunction in women, including general health status, psychological disorders, chronic diseases, individual and social factors [6]. Also, other factors such as urinary incontinence [7], metabolic syndrome [8], chronic renal failure [9], substance abuse [10], and some behavioral disorders [11] may affect sexual function in women.

Iron deficiency anemia is one of the most common forms of malnutrition. Thirty to fifty percent of worldwide anemia is attributed to iron deficiency [12]. Almost 1.6 billion people globally are suffering from anemia most of whom have iron deficiency anemia [13]. The prevalence of anemia among women in Turkey is 27.8% that 56% of them have iron deficiency anemia (IDA) [14]. According to a study among women aged 15–45 in Zanjan, Iran, 23.6% of women had anemia [15]. IDA causes symptoms such as weakness, headache, restlessness, fatigue, anxiety, pallor, palpitation, reduced strength, impair learning and productivity, and reduced physical and mental capacity [16–17]as well as fatigue, poor mental health, lack of concentration and poor pregnancy outcomes [18]. Since IDA can cause anxiety and fatigue in women, and these can, in turn, be effective factors in sexual function, the IDA could be considered as a factor to reduce sexual function. [18–19].

A study by Gulmez et al. [20] showed that after treatment of IDA in women, most of the sexual function domains were significantly enhanced. Also, studies showed that with increasing serum hemoglobin level, sexual performance score, level of energy, physical and social function increased, and anxiety and depression score decreased, that all of these factors are related to better sexual function [21–22].

In contrast, there are other studies that have reached contradictory results. The results of a study showed that Hb levels in women with and without sexual dysfunction were not significantly different [23]. Also studies showed that women with hypothyroidism have a lower sexual function in comparison to normal women [24–25].

Given the paucity of studies on the era, the hypothesis of the study was: is there a relationship between iron deficiency anemia and sexual function and satisfaction among reproductive-aged women in Iran.

## Materials and methods

This study is a matched cohort study to determine the association of IDA with sexual satisfaction and function in 129 women (52 with IDA and 77 non-IDA) of reproductive age in Mahshar, Iran. Eligible women were recruited from a public health clinic in Mahshar from September to June 2017.

The inclusion criteria were as follows: age 18–45 years, married for more than one year, sexually active, having basic literacy, and being monogamous. Women with chronic diseases, pregnancy, breastfeeding, early menopause, mental illness and depression or infertility, drug addiction, alcohol consumption or smoking and use of medications that had an effect on sexual function were excluded from the study.

Based on a pilot study to detect a 12-point difference at the primary outcome (the sexual satisfaction) with 12, 27.12 as respectively standard deviations of IDA and non-IDA and 5% level of significance and 90% power and 10% non- responsiveness, it was estimated that 52 participants would be necessary for each group.

To select the subjects, the researcher (ZN) attended to one public health clinic in Mahshar and explained the aims of the study to the eligible women and asked those who were willing to participate to complete the questionnaires.

### Measurements

A socio-demographic questionnaire was used to gather information regarding the demographic characteristics of the participants.

Female Sexual Functions Index (FSFI) was used to gather data regarding sexual function. The FSFI consists of 19 questions (rated from 0 to 5) and 6 domains, including desire, arousal, lubrication, orgasm, satisfaction, and pain. In order to calculate the individual domain score, the scores of individual questions comprising the domain were summed and multiplied by the factor specific to the relevant domain. The total score is calculated by adding the scores of the six domains. The total score of the scale ranges from 2 to 36 with higher scores indicating a better degree of sexual function. The validity and reliability of the original and the Persian versions of this questionnaire have been assessed and confirmed by Rosen et al. [26] and Fakhri et al. [27] respectively.

The level of sexual satisfaction was measured using Larson Sexual Satisfaction (LSS). LSS has 25 questions scored from one to five according to the Likert Scale. Score one represents *never* and five represents *always*. Sexual satisfaction was classified into four sections of sexual dissatisfaction (≤50), low sexual satisfaction (51–75), average sexual satisfaction (76–100), and high sexual satisfaction (> 101). The validity and reliability of the original and the Persian versions of LSS have been approved by Larson et al. [28] and Bahrami et al. [29] respectively.

For measuring the hematologic status of participants, 5 mL venous blood was taken [15]. Two milliliters of blood was mixed with citrate for measuring Hb, and HCT, while three ml was used to separate the sera. The blood and sera were sent to a reference laboratory in the hospital for measuring blood indices including hemoglobin, hematocrit, serum iron, and ferritin. In this study, serum ferritin level was measured using ELISA method.

**Fig 1:**
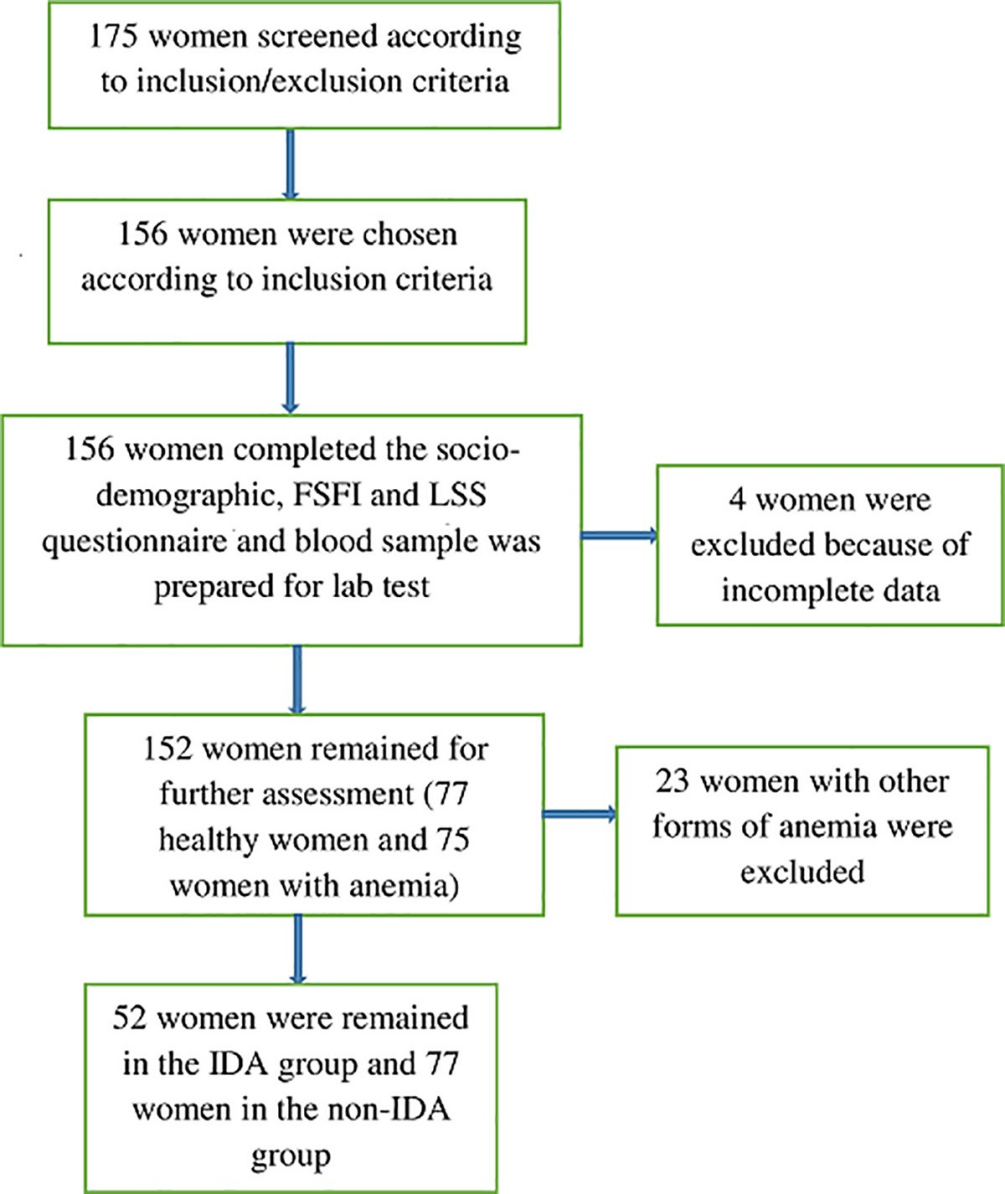
Flowchart of recruitment and retention of participants in the study.

In this study, 175 women were screened according to the inclusion and exclusion criteria and 156 women had inclusion criteria. According to the results of blood tests, 52 women had IDA and matched with 77 women in the non-IDA group in terms of age, education level and contraceptive methods (Fig 1). Participants in the case and control groups were requested to complete a socio-demographic questionnaire, FSFI and LSS questionnaires. One of the researchers (ZN) was available to resolve any ambiguity.

#### Ethical consideration

The design of the study was approved by the Ethics Committee of Ahvaz Jundishapur University of Medical Sciences (Ref No: IR.AJUMS.REC.1395.219). All participants gave written informed consent prior to data collection.

### Statistical analysis

Statistical analysis was carried out using SPSS version 19. Quantitative data were computed as means and standard deviations (SD), and qualitative data were presented as frequencies and percentages. Based on normality, the data distribution was analyzed using an independent t-test or Mann-Whitney tests. The Chi-square test was used to compare qualitative variables between the two groups. In this study, the assumptions of Pearson correlation did not meet, so non-parametric correlation coefficient was used to test the relationship between blood indices and sexual function and satisfaction. P values less than 0.05 were considered significant.

## Results and discussion

A total of 156 women were recruited for this study and 129 completed the study. The flowchart of recruitment and retention of participants is presented in Fig 1.

The socio-demographic characteristics of participants are presented in Table 1. There was no significant difference between the two groups in any of the baseline characteristics.

**Table 1:** Socio-demographic and reproductive characteristic of the responders.

Characteristic		IDA group (n = 52)	Non-IDA group (n = 77)	P-value
		Mean ±SD or N (%)		
Marriage duration (y)*		12.26 ±6.42	10.68 ±6.02	0.36
Age (y)*		33.23±5.25	32.33±5.2	0.52
Spouse’s Age (y)*		38.07±6.85	37.67±5.7	0.93
Educational level	Elementary	10 (19.2)	10 (13)	0.64
	Secondary	11 (21.2)	19 (24.7)	
	Diploma	21 (40.4)	28 (36.4)	
	Academic	10 (19.2)	20 (27)	
Spouse’s Education	Elementary	8 (15.4)	13 (16.9)	0.93
	Guidance	16 (30.8)	21 (27.3)	
	Diploma	18 (34.6)	25 (32. 5)	
	College	10 (19.3)	18 (23.4)	
Job	Housewife	48 (92.3)	63 (81.8)	0.92
	Employed	4 (7.7	14(18.2)	
Spouse’s job	Self- Employment	25 (48.1)	45 (58.4)	0.31
	Employed	23 (44.2)	29 (37.7)	
	Unemployed	4 (7.7)	3 (3.9)	
Economic status	Good	4 (7.7)	12 (56.4)	0.36
	Moderate	39 (75)	55 (71.4)	
	Poor	9 (17.3)	10 (13)	
Number of children*		2.11±1.21	1.84±1.06	0.17
Type of previous delivery	NVD	22 (42.3)	40 (51.9)	0.34
	C/S	25 (48.0)	34 (44.1)	
	NVD+C/S	1 (1.9)	0 (0)	
	Nulliparous	4 (7.6)	3 (3.8)	
Contraception	OCP	8 (15.4)	14 (18.2)	0.92
	Condom	13 (25)	19 (24.7)	
	IUD	5 (9.6)	11 (14.3)	
	TL	2 (3.8)	2 (2.6)	
	Withdrawal	22 (42.3)	27 (35.1)	
	No methods	2 (3.8)	4 (5.2)	
Menstruation	Regular	41 (78.8)	65 (84.4)	0.92
	Irregular	11 (21.2)	12 (15.6)	

* Mean (SD); the data are given as N (%), C/s: Cesarean section, NVD: Normal Vaginal Delivery, OCP: Oral

Contraceptive pills, IUD: Intra Uterine Device, TL: Tubal Ligation

Most women in the two groups had one or two children. Most women in both groups had regular menstrual cycles. There was no significant difference between the groups in terms of reproductive characteristics (Table 1).

The means of Hb, HCT, serum iron and ferritin in the IDA group was significantly lower than those in the non-IDA group (p<0.01, Table 2). The scores of desire, arousal, lubrication, orgasms, and satisfaction were significantly lower in women with anemia compared to the normal women (p<0.001). Also, the IDA group had significantly more pain than that in the non-IDA group (3.48 ±1.50 vs. 4.29 ±1.25, p<0.001). The total score of sexual function and sexual satisfaction in the IDA group was significantly lower than that in the non-IDA group (19.76 ±5.40 vs. 26.76 ±3.91, p<0.001) and (85.28± 20.24 vs. 99.16 ±16.73, p<0.001) respectively (Table 3).

**Table 2:** Comparison of mean and standard deviation of blood indices in IDA and non-IDA groups.

Variable	IDA group (n = 52)	Non-IDA group (n = 77)	Confidence interval (95%)	P-value
	Mean ± SD			
Hb (g/dL)	10.84 ±0.65	12.66 ±0.43	1.62–2	<0.001
HCT(%)	34.50 ±1.57	38.22 ±1.04	3.26–4.17	<0.001
Serum iron (μg/dL)	22.04 ±6.20	75.94 ±30.01	45.4–62.34	<0.001
Ferritin (μg/L)	10.23 ±7.53	61.24 ±87.08	26.7–75.23	<0.001

95% CI is about around the mean difference

**Table 3:** Sexual function and satisfaction scores in IDA and non-IDA groups.

Sexual function	IDA group (n = 52)	Non-IDA group (n = 77)	P-value
	Mean ±SD or N(%)		
Desire	2.57 ±0.97	3.64 ±0.85	<0.001
Arousal	2.87 ±1.07	4.06 ±0.89	<0.001
Lubrication	3.64 ±1.38	4.80 ±0.87	<0.001
Orgasm	3.33 ±1.57	4.91 ±0.97	<0.001
Satisfaction	3.72 ±1.28	5.04 ±0.97	<0.001
Pain	3.48 ±1.50	4.29 ±1.25	<0.001
**Total Sexual function**	19.76 ±5.40	26.76 ±3.91	<0.001
Larsson Sexual Satisfaction, mean ±SD	85.28± 20.24	99.16 ±16.73	<0.001
Dissatisfaction with sex (0–50)	2 (3.84)	1 (1.29)	<0.001
Low sexual satisfaction (51–75)	11 (21.15)	4 (5.19)	
Medium sexual satisfaction (76–100)	30 (57.6)	28 (36.3)	
High sexual satisfaction (Score > 100)	9 (17.3)	44 (57.14)	

As evident in Table 4, all blood indices for IDA had a significant relationship with all sexual function components and sexual satisfaction (p = 0.01) except for pain with HB and ferritin.

This study aimed to evaluate the relationship between iron deficiency anemia and sexual function and satisfaction among reproductive-aged Iranian women. Our results showed that women with iron deficiency anemia had lower scores of sexual function and satisfaction.

**Table 4:** The relationship of sexual function components with blood indices for IDA.

Blood indices	Desire	Arousal	Lubrication	Orgasm	Sexual Satisfaction	Pain	LSS score	Total score of FSFI
HB (g/dl)	0.42**	0.46**	0.36**	0.48**	0.43**	0.15	0.33**	0.55**
HCT (%)	0.41**	0.40**	0.38**	0.42**	0.42**	0.16**	0.35**	0.47**
Ferritin (μg/L)	0.41**	0.50**	0.32**	0.43**	0.47**	0.12	0.34**	0.52**
Serum iron (μg/dL)	0.48**	0.49**	0.34**	0.42**	0.46**	0.26**	0.38**	0.53**

** Significant at 0.01

Iron deficiency anemia is the most common type of anemia and is a public health problem that affects populations in both rich and poor countries. Because of the high demand for iron during a pregnancy, lactation, menstruation, and nutritional deficiencies, IDA remained the most common cause of iron deficiency in reproductive age women. According to the WHO, the global prevalence of anemia is 24.8% that is about 1.62 billion people worldwide. However, among different population groups, the greatest number of individuals (468.4 million) affected by anemia belongs to non-pregnant women [30–31]. A study showed that the prevalence of anemia and IDA was 33% and 16.6%, respectively, for reproductive age women in urban and rural areas of Iran [32].

The evidence indicated that anemia is one of the important factors related to mental well-being and quality of life [17], however, scant data regarding the impact of anemia on sexual function and satisfaction is available.

The results of this study indicated that all areas of sexual function were significantly impaired in anemic women. Results of other studies also indicated an impaired sexual function in women with iron deficiency anemia. A prospective study was conducted by Gulmez et al., [20] in Turkey which aimed to assess sexual performance and quality of life in women with iron deficiency anemia before and after treatment. There was a significant difference between pre- and post-treatment in level of hemoglobin, hematocrit, serum iron, and serum iron-binding capacity. Beck Anxiety Inventory (BAI) scores decreased and FSFI scores significantly increased after IDA treatments (P< 0.001). Our results in terms of the relationship between IDA and sexual function are similar to Gulmez et al.

The underlying cause of female sexual dysfunction in women with IDA is not fully understood. However, some studies revealed that women with IDA have more hypothyroidism [33] and hypothyroidism, in turn, can deteriorate the sexual function. Results of Pasquali et al.’s study showed that the prevalence of female sexual dysfunction in women with hypothyroidism was 46.1% in comparison with 20.7% in the control group [25]. Furthermore, depression and anxiety are both contributed to sexual function may happen more in the basis of IDA and a study showed that with the treatment of IDA the scores of depression decreased and sexual function also improved significantly [20].

Beside thyroid gland, another underlying cause for sexual dysfunction among women with IDA is a decrease of sex hormones. A cross-sectional study with the aim of determining the relationship between serum ferritin and sexual hormones by Liu et al., was done in China [34]. Results showed that ferritin level had a significant and an inverse relationship with total serum testosterone, sex hormone-binding protein (SHBG) and free testosterone. However, there was only a slight non-significant negative correlation between ferritin and estradiol levels.

Some studies have shown that low testosterone levels are associated with low hemoglobin levels, and testosterone deficiency can reduce sexual desire and satisfaction. Also, there is some evidence that testosterone supplementation can normalize the iron status and improve the anemia in older mice [35]. Ekart et al. in their study on hemodialysis patients, found a correlation between serum testosterone concentration and hemoglobin [36]. Some previous studies showed an association between testosterone levels and hemoglobin in non-hemodialysis patients. Bhatia et al. demonstrated that both low testosterone and chronic inflammation contributed to mild anemia in type-2 diabetic men [37]. Gebremedhin & Enquselassie found that testosterone deficiency contributed to an increased frequency of anemia in men with type-2 diabetes mellitus [31].

Our results indicated that all blood indices for IDA had a significant relationship with all sexual function dimensions and sexual satisfaction except for pain that did not show any significant relationship with HB and ferritin. We could not find any study that evaluates the relationship of blood indices in IDA and sexual function. However, Teuwafeu et al., in their study on women who underwent maintenance hemodialysis found that there is a significant relationship between anemia and sexual function [38].

### Strengths and limitations of the study

To the best of our knowledge, this is the first study examining the relationship between iron deficiency anemia with sexual satisfaction and function in women of reproductive age. Women enrolled from a public health center that this center providing care for the whole population and not just the sick people.

This study has some limitations that are worth mentioning. First, this study was done on only women in the south of Iran; therefore, the findings cannot be applied to other women or girls in Iran. Another limitation of the study is using self-reporting questionnaires. In this study, we did not assess the sexual function of male partners; therefore, further study in men could provide comprehensive information in this area.

### Conclusion

The results of this study showed that the score of all dimensions of sexual function in anemic women is lower than that in healthy women. Therefore, screening, education, and counseling about anemia and sexual function among anemic women in reproductive age are recommended. Also, it is recommended that iron deficiency anemia should be considered as an effective factor in treating a patient with sexual dysfunction and sexual dissatisfaction.

## References

[1] American Psychiatric Association, “Appendix. Highlights of changes from DSM-IV to DSM-5,” in Diagnostic and Statistical Manual of Mental Disorders, American Psychiatric Association, Arlington, Va, USA, 5th edition, 2013.

[2] Lewis RW, Fugl‐Meyer KS, Corona G, Hayes RD, Laumann EO, Moreira ED Jr, et al Original articles: definitions/epidemiology/risk factors for sexual dysfunction. J Sex Med 2010; 7: 1598–607. 10.1111/j.1743-6109.2010.01778.x 20388160 10.1111/j.1743-6109.2010.01778.x

[3] Laumann EO, Paik A, Rosen RC. Sexual dysfunction in the United States: prevalence and predictors. JAMA 2000; 281: 537–44. 10.1001/jama.281.6.537 10022110

[4] Safarinejad MR. Female sexual dysfunction in a population-based study in Iran: prevalence and associated risk factors. Int J Impot Res 2006;18: 382–95. 10.1038/sj.ijir.3901440 16395324 10.1038/sj.ijir.3901440

[5] Naeinian MR, Shaeiri MR, Hosseini FS. General Health and Quality of Life in Patients With Sexual Dysfunctions. Urology Journal 2011; 8:127–131. 21656471

[6] Dunn KM, Croft PR, Hackett GI. Association of sexual problems with social, psychological, and physical problems in men and women: a cross-sectional population survey. J Epidemiol Community Health 1999; 53:144–148. 10396490 10.1136/jech.53.3.144 PMC1756846

[7] Salonia A, Zanni G, Nappi RE, Briganti A, Dehò F, Fabbri F, et al Sexual dysfunction is common in women with lower urinary tract symptoms and urinary incontinence: results of a cross-sectional study. Eur Urol 2004; 45: 642–8. 10.1016/j.eururo.2003.11.023 15082208 10.1016/j.eururo.2003.11.023

[8] Esposito K, Ciotola M, Marfella R, Di Tommaso D, Cobellis L, Giugliano D. The metabolic syndrome: a cause of sexual dysfunction in women. Int J Impot Res 2005;17: 224–6. 10.1038/sj.ijir.3901310 15716979 10.1038/sj.ijir.3901310

[9] Palmer BF. Sexual dysfunction in men and women with chronic kidney disease and end-stage kidney disease. Adv Ren Replace Ther 2003;10: 48–60. 10.1053/jarr.2003.50003 12616463 10.1053/jarr.2003.50003

[10] Diehl A, Da Silva RL, Laranjeira R. (2013). Female sexual dysfunction in patients with substance-related disorders. Clinics (Sao Paulo) 2013;68: 205–211. 10.6061/clinics/2013(02)OA14 23525317 10.6061/clinics/2013(02)OA14 PMC3584261

[11] Salehzadeh M, Kajbaf MB, Molavi H, Zolfaghari M. Effectiveness of cognitive–behavior therapy on sexual dysfunction in women. Psychological Studies 2011; 7: 11–13

[12] Conclusions and recommendations of the WHO Consultation on prevention and control of iron deficiency in infants and young children in malaria-endemic areas. World Health Organization. Food Nutr Bull. 2007 12; 28(4 Suppl): S621–7. 18297899 10.1177/15648265070284s414

[13] McLean E, Cogswell M, Egli I, Wojdyla D, de Benoist B. (2009). Worldwide prevalence of anaemia, WHO Vitamin and Mineral Nutrition Information System, 1993–2005. Public Health Nutr 2009; 12: 444–54. 10.1017/S1368980008002401 18498676

[14] Saydam BK, Genc RE, Sarac F, Turfan EC. Prevalence of anemia and related factors among women in Turkey. Pak J Med Sci. 2017 Mar-Apr; 33(2): 433–438. doi: 10.12669/pjms.332.11771 28523051 10.12669/pjms.332.11771 PMC5432718

[15] Bateni J, Shoghli A. The Prevalence of Iron Deficiency Anemia (IDA) Based on Hematologic Indices in Non-pregnant Women Aged 15–45 in Zanjan. ZUMS Journal 2006; 14: 39–46

[16] Nowrousian MR. Impact of anemia and red blood cell transfusion on organ function In: Nowrousian M.R. (eds) Recombinant Human Erythropoietin (rhEPO) in Clinical Oncology. Springer, Vienna 2008.

[17] Fretham SJB, Carlson ES, Georgieff MK. (2011). The Role of Iron in Learning and Memory. Adv Nutr 2011; 2: 112–121. 10.3945/an.110.000190 22332040 10.3945/an.110.000190 PMC3065765

[18] Mawani M, Aziz Ali S, Bano G, Aziz Ali S. Iron Deficiency Anemia among Women of Reproductive Age, an Important Public Health Problem: Situation Analysis. Reprod Syst Sex Disord 2016;5:187 10.4172/2161-038X.1000187

[19] Bradford A, Meston CM. The impact of anxiety on sexual arousal in women. Behav Res Ther 2006; 44: 1067–1077. 10.1016/j.brat.2005.08.006 16199003 10.1016/j.brat.2005.08.006 PMC2857771

[20] Gulmez H, Akin Y, Savas M, Gulum M, Ciftci H, Yalcinkaya S, Yeni F. Impact of iron supplementation on sexual dysfunction of women with iron deficiency anemia in short term: a preliminary study. J Sex Med 2014;11:1042–6. 10.1111/jsm.12454 24754331 10.1111/jsm.12454

[21] Asadifard F, Zeighami Mohammadi S, Bahrami Baba Heydari T. Sexual function of women with Chronic Renal Failure Undergoing Hemodialysis and factors related to it. IJCCN 2013;5(4):204–14.

[22] Peuranpää P, Heliövaara-Peippo S, Fraser I, Paavonen J, Hurskainen R. Effects of anemia and iron deficiency on quality of life in women with heavy menstrual bleeding. Acta Obstet Gynecol Scand. 2014 7;93(7):654–60. 10.1111/aogs.12394 24912842 10.1111/aogs.12394

[23] Patterson AJ, Brown WJ, Powers JR, Roberts DC. Iron deficiency, general health and fatigue: results from the Australian Longitudinal Study on Women's Health. Qual Life Res 2000; 9: 491–7 11190004 10.1023/a:1008978114650

[24] Maldonado-Araque C, Valdes S, Lago-Sampedro A, Antonio Lillo-Muñoz J, Garcia-Fuentes E, Perez-Valero V, et al Iron deficiency is associated with Hypothyroxinemia and Hypotriiodothyroninemia in the Spanish general adult population: Di@bet.es study. SCIENTIFIC RePorts | (2018) 8:6571 | 10.1038/s41598-018-24352-9 29700318 10.1038/s41598-018-24352-9 PMC5919900

[25] Pasquali D, Maiorino MI, Renzullo A, Bellastella G, Accardo G, Esposito D. Female sexual dysfunction in women with thyroid disorders. J Endocrinol Invest. 2013 10;36(9):729–33. 10.3275/8933 23580027 10.3275/8933

[26] Rosen R, Brown C, Heiman J, Leiblum S, Meston CM, Shabsigh R, et al (2000). The Female Sexual Function Index (FSFI): A multidimensional self-report instrument for the assessment of female sexual function. J Sex Marital Ther 2000; 26:191–208. 10.1080/009262300278597 10782451 10.1080/009262300278597

[27] Fakhri A, Pakpour AH, Burri A, Morshedi H, Zeidi IM. The Female Sexual Function Index: translation and validation of an Iranian version. J Sex Med 2012; 9: 514–23. 10.1111/j.1743-6109.2011.02553.x 22146084 10.1111/j.1743-6109.2011.02553.x

[28] Larson JH, Anderson SM, Holman TB, Niemann BK. A longitudinal study of the effects of premarital communication, relationship stability, and self-esteem on sexual satisfaction in the first year of marriage. J Sex Marital Ther 1998; 24:193–206. 10.1080/00926239808404933 9670124 10.1080/00926239808404933

[29] Bahrami N, Yaghoob Zadeh A, Sharif Nia H, Soliemani MA, Haghdoost AA. Validity and Reliability of the Persian Version of Larson sexual Satisfaction Questionnaire in Couples. J Kerman Uni Med Sci 2016; 23: 344–356

[30] de Benoist B, McLean E, Egli I, Cogswell M. Worldwide prevalence of anaemia 1993–2005: WHO global database on anaemia. Geneva: World Health Organization 2008.

[31] Gebremedhin S, Enquselassie F. Correlates of anemia among women of reproductive age in Ethiopia: Evidence from Ethiopian DHS 2005. Ethiop J Health Dev 2011;25: 22–30.

[32] Sheikholeslam R, Jamshidbeygi E, Salehian P, Malekafzali H. (2001). Prevalence of iron deficiency, anemia and iron deficiency anemia in reproductive age women (49–15 years) in urban and rural areas of Iran. Teb VA Tazkiyeh 2001; 47: 37–44.

[33] Li S, Gao X, Wei Y, Zhu G, Yang C. The Relationship between Iron Deficiency and Thyroid Function in Chinese Women during Early Pregnancy. J Nutr Sci Vitaminol (Tokyo). 2016;62(6):397–401. 10.3177/jnsv.62.397 28202844 10.3177/jnsv.62.397

[34] Liu Z, Ye F, Zhang H, Gao Y, Tan A, Zhang S, Xiao Q, et al The association between the levels of serum ferritin and sex hormones in a large scale of Chinese male population. PLoS One. 2013 10 11;8(10):e75908 10.1371/journal.pone.0075908 24146788 10.1371/journal.pone.0075908 PMC3795691

[35] Guo W, Li M, Bhasin S. Testosterone Supplementation Improves Anemia in Aging Male Mice. J Gerontol A Biol Sci Med Sci 2013; 69:505–13. 10.1093/gerona/glt127 23974081 10.1093/gerona/glt127 PMC3991143

[36] Ekart R, Taskovska M, Hojs N, Bevc S, Hojs R. Testosterone and Hemoglobin in Hemodialysis Male and Female Patients. Artif Organs 2014; 38: 588–603. 10.1111/aor.12218 24256140

[37] Bhatia V, Chaudhuri A, Tomar R, Dhindsa S, Ghanim H, Dandona P. (2006). Low testosterone and high C-reactive protein concentrations predict low hematocrit in type 2 diabetes. Diabetes Care 2006;29: 2289–94. 10.2337/dc06-0637 17003308 10.2337/dc06-0637

[38] Teuwafeu D. Ashuntantang G. Essi MJ, Kaze F, Maimouna M, Balepna J, et al Sexual function and correlates in women undergoing maintenance hemodialysis in Cameroon: a multi-centric study. Nephrology Dialysis Transplantation. 2016;31(1): i295–i296. 10.1093/ndt/gfw175.46

